# Genome-Wide Identification, Characterization, and Expression Profiles of *TLR* Genes in Darkbarbel Catfish (*Pelteobagrus vachelli*) Following *Aeromonas hydrophila* Infection

**DOI:** 10.3390/biology14121724

**Published:** 2025-12-01

**Authors:** Zhengyong Wen, Lisha Guo, Jianchao Chen, Qiyu Chen, Yanping Li, Yunyun Lv, Qiong Shi, Shengtao Guo

**Affiliations:** 1 Fishes Conservation and Utilization in the Upper Reaches of the Yangtze River Key Laboratory of Sichuan Province, College of Fisheries, Neijiang Normal University, Neijiang 641100, China; zhengyong_wen@126.com (Z.W.); guolisha2018@163.com (L.G.); liyanping_sci@foxmail.com (Y.L.); lvyunyun_sci@foxmail.com (Y.L.); 2 Laboratory of Aquatic Genomics, College of Life Sciences and Oceanography, Shenzhen University, Shenzhen 518057, China; 2410173010@mails.szu.edu.cn (J.C.); qyuchen2025@163.com (Q.C.); 3 College of Life Sciences, Neijiang Normal University, Neijiang 641100, China

**Keywords:** darkbarbel catfish, Toll-like receptor, evolutionary traits, immune response

## Abstract

Toll-like receptors (TLRs) are central to pathogen recognition in teleost innate immunity. In this study, we surveyed 41 genomes from four representative teleost orders (i.e., Cypriniformes, Siluriformes, Perciformes, and Pleuronectiformes) for 15 *TLR* genes (*TLR*1–9, 12, 13, 18, 20–22) revealed a conserved core (*TLR*2/3/7 in nearly all examined species) alongside lineage-specific losses (*TLR*4/9/18/20/21/22), indicating both strong conservation and dynamic diversification of the *TLR* repertoire. We further identified and characterized 12 *TLR* genes in economically important darkbarbel catfish (*Pelteobagrus vachellii*). Corresponding cDNAs span 2089–4456 bp and encode proteins of 789–1,087 aa, with canonical extracellular LRR arrays and C-terminal TIR domains but notable “non-classical” features (such as absence of signal peptides in TLR1/13; no transmembrane segment in TLR7; multiple transmembranes in TLR3/8/13/18/22), suggesting subcellular and functional heterogeneity of various *TLR* genes. Subsequent gene-structure comparisons uncovered gene-specific exon–intron organizations and variable UTR lengths, implicating differential post-transcriptional regulation. Predicted 3D structures retain the traditional hallmark LRR horseshoe fold with subtle variations potentially tuning ligand specificity. Genomic synteny with *Pseudobagrus ussuriensi* and *Pangasianodon hypophthalmus* reveals conserved chromosomal organization, and phylogeny construction resolves each TLR subtype into well-supported monophyletic clades, which underscore evolutionary stability. Functionally, exogenous *Aeromonas hydrophila* challenge triggered rapid, tissue-dependent *TLR* up-regulation in the kidney, liver, and especially gill (with some transcripts > 1000-fold), highlighting coordinated mucosal and systemic surveillance in darkbarbel catfish. Taken together, these valuable data provide a comprehensive framework for the structural, evolutionary, and inducible expression landscape of catfish *TLR*s and establish a foundation for in-depth studies on antibacterial immunity in diverse teleost species.

## Simple Summary

Fish farmers face growing losses from bacterial diseases, yet we still know too little about how many pathogen-sensing genes fish carry and how they respond during infection. In this study, we examined the family of “Toll-like receptors”, the immune system’s early-warning sensors for invading microbes, across many fish species and then focused on the representative darkbarbel catfish, an important farmed species in China. We found twelve such receptor genes in this catfish and showed that they share a common blueprint but differ in the details that likely tune what each detects. When healthy fish were exposed to *Aeromonas hydrophila*, a common disease-causing bacterium, these genes switched on quickly and differently in the kidney, liver, and especially the gill, pointing to a powerful first line of defense at the body’s surfaces. Our results map the main immune sensors in this species and reveal when and where they act during infection. This knowledge can guide breeding, vaccine design, and farm practices to reduce disease losses and limit the need for antibiotics, supporting more sustainable aquaculture.

## 1. Introduction

Darkbarbel catfish (*Pelteobagrus vachelli*), an economically significant species in the Yangtze River basin, has been valuable for its rapid growth, hardiness, and natural resistance to various pathogens [1]. It also plays a pivotal role as a male parent in cross-breeding programs, notably contributing to the creation of the fast-growing, disease-tolerant “Huangyou 1” hybrid with yellow catfish *Peltobagrus fulvidraco* [2]. However, the shift towards high-density and intensive farming has raised concerns regarding genetic diversity loss and increasing prevalence of infectious diseases in cultured populations [1]. Among the most harmful pathogens, *Edwardsiella ictaluri* often causes “Red Head Disease” that can lead to up to 50% mortality [3], and *Aeromonas hydrophila* is usually responsible for ulcerative “Rotting Body Disease” and systemic septicemia [4]. Therefore, understanding the populations’ genetic structure of *P. vachelli* and its molecular responses to these exogenous pathogens is vital for protecting broodstock resources and developing effective disease-control strategies.

Teleost possess both innate and adaptive immune systems, although their defense mechanisms are primarily reliant on the innate immunity, as the adaptive response develops more slowly with a narrower repertoire diversity [5]. The innate immune system consists of physical barriers, immune cells, and soluble factors that work together to enable the rapid recognition and elimination of pathogens [6]. A key component of this early recognition is pattern-recognition receptors (PRRs), which detect conserved pathogen-associated molecular patterns (PAMPs) and initiate antimicrobial responses [7]. Toll-like receptors (TLRs), a well-studied and evolutionarily conserved family of PRRs, are central to this process in vertebrates [8,9]. By recognizing a broad spectrum of microbial ligands, TLRs trigger signaling cascades, primarily through NF-κB and related transcription factors to regulate pro-inflammatory and antiviral gene expression [8]. Teleost possess both ancestral and species-specific TLRs, such as TLR21-TLR23, reflecting lineage-specific expansions likely adapted to the aquatic pathogen environment [10]. These interesting characteristics highlight the pivotal role of TLRs in pathogen detection and immune activation, providing a molecular foundation for developing effective disease prevention strategies in aquaculture practices [11,12,13].

As lower vertebrates, fishes possess both innate and adaptive immune systems, with the innate branch serving as the primary defense against invading pathogens [6]. Within this branch, the TLR family is the central group of PRRs that detect conserved PAMPs such as bacterial lipopolysaccharide [14] and viral double-stranded RNA [8,9]. Genomic and functional studies demonstrate that piscine TLRs are evolutionarily conserved, with some members (such as TLR2, TLR4, and TLR5) identified in numerous teleost species including *P. vachelli* [9]. Ligand binding activates canonical signaling pathways, particularly the MyD88-dependent NF-κB pathway, leading to transcription of pro-inflammatory cytokines and interferons that coordinate antimicrobial responses [8]. Recent molecular studies in *P. vachelli* show that pvTLR5 exists in both membrane-bound and soluble isoforms, with its transcription significantly upregulated following *A. hydrophila* challenge [15]. High-throughput spleen transcriptomics further revealed that TLR22, a teleost-specific TLR, is one of the most highly induced receptors during bacterial infection, highlighting its role in front-line defense [4]. These findings highlight the critical role of TLR signaling in the immune defense of *P. vachelli*, and provide a mechanistic framework for developing immunomodulatory strategies to combat diseases in intensive aquaculture.

TLRs, as core PRRs of the innate immune system, serve as the first molecular barrier against pathogen invasion in teleost [8,9]. However, a comprehensive genome-wide characterization of various *TLR* genes and a detailed understanding of their antibacterial regulatory network in *P. vachelli* remain lacking. Although the recently released chromosome-level genome provides a valuable foundation for such studies [1], functional insights are primarily derived from single-gene investigations, such as the findings of pv*TLR5* that was upregulated following *A. hydrophila* infection [15], or bulk-spleen transcriptomes that capture broad immune signatures without resolving individual TLR pathways [4]. Consequently, key questions remain unknown regarding the evolutionary diversification of the full TLR repertoire, their tissue- and stimulus-specific expression profiles, and the molecular mechanisms underlying TLR-mediated recognition of *A. hydrophila* in *P. vachelli*. Resolution of these issues, in fact, needs a systematic genome-to-function approach.

Despite the foundational knowledge of TLRs in immune responses, several significant gaps exist in understanding the TLR-mediated immunity in *P. vachelli*. First, the complete genomic landscape of *TLR* genes in this species remains underexplored, leaving the evolutionary history and full repertoire of TLRs largely unresolved. Previous studies have focused on individual genes, but a comprehensive analysis on the entire *TLR* family, their gene structure, and their phylogenetic relationships has not been conducted. Second, while the immune response to *A. hydrophila* has been investigated in a broader sense, the specific expression patterns of different *TLR* members in response to infection have not been elucidated in detail. This gap in understanding the tissue- and stimulus-specific expression of *TLR*s hinders a clear comprehension of their roles in antibacterial immunity. Addressing these issues will provide critical insights into the TLR-mediated immune system in *P. vachelli*, which has not been fully characterized in any teleost species.

Our present study aims to comprehensively identify the *TLR* gene family in *P. vachelli* through whole-genome analysis, and to explore their immune responses to *A. hydrophila* infection. The research will analyze the gene structure, protein spatial configuration, and phylogenetic relationships of the *P. vachelli* TLR family. Additionally, transcriptional analysis will reveal the expression patterns of different *TLR* members after the *A. hydrophila* infection, elucidating the molecular mechanisms by which key *TLR* genes contribute to antibacterial immunity. These findings will not only fill some theoretical gaps in understanding the innate immune system of *P. vachelli*, but also provide novel molecular targets and immune regulatory strategies for preventing and controlling bacterial diseases in this economic fish species. These insights are expected to offer practical guidance for sustainable development of the *P. vachelli* aquaculture industry.

## 2. Materials and Methods

### 2.1. Genome-Wide Identification of TLR Genes in P. vachellii

To comprehensively identify the *TLR* gene family in *P. vachelli*, a representative set of species spanning diverse evolutionary lineages was selected. These fishes included *Leucoraja erinacea* (little skate), *Lepisosteus oculatus* (spotted gar), *Danio rerio* (zebrafish), *Ctenopharyngodon idella* (grass carp), *Cirrhinus molitorella* (mud carp), *Chanodichthys erythropterus* (predatory carp), *Cololabis saira* (Pacific saury), *P. vachelli*, *Astyanax mexicanus* (Mexican tetra), *Triplophysa tibetana* (stone loach), *Protopterus annectens* (west African lungfish), and *Lepidosiren paradoxa* (south American lungfish). This broad phylogenetic framework was used to analyze the diversity and evolution of the *TLR* gene family.

Protein sequences from these selected species were retrieved from the NCBI database (https://www.ncbi.nlm.nih.gov/, accessed on 20 July 2024), and orthologous gene clustering was performed using OrthoFinder (v2.5.4) with the following optimized parameters: -S diamond -t 100 -M msa -A mafft -T iqtree. OrthoFinder is a sequence similarity-based tool designed to identify orthologous gene families across various species and reconstruct phylogenetic relationships. This analysis established orthologous relationships for *TLR* genes across these examined species.

To further identify TLR family members, BLAST (http://www.ncbi.nlm.nih.gov/blast accessed on 20 July 2024) searches were conducted against the publically available genomic data of these selected species (Table 1), using an E-value threshold of 1 × 10^−5^ to ensure high specificity. The combination of OrthoFinder results and BLAST searches allowed for confident identification of diverse *TLR* genes in each species, facilitating subsequent sequence comparisons. Finally, application of the HMMER tool [16] for HMM validation of the identified *TLR* genes further confirmed accuracy and completeness of these genes, ensuring a comprehensive identification of *TLR* family members.

To verify the completeness and functionality of the identified *TLR* genes, their domain architecture was analyzed using the SMART (Simple Modular Architecture Research Tool; http://smart.embl-heidelberg.de/, accessed on 20 July 2024). The functional integrity of deduced TLR proteins depends on the coordinated presence of three key domains, including an extracellular leucine-rich repeat (LRR) domain for pathogen recognition, a transmembrane domain for membrane anchoring, and an intracellular TIR domain for downstream signal transduction. Sequences lacking any of these domains, particularly those containing only the TIR domain, were excluded as they are functionally deficient without full length. Only those sequences with the complete set of LRR, transmembrane, and TIR domains were retained for further analysis. For the 3D structure prediction, we employed AlphaFold for protein structure modeling. The prediction results were validated using TM-score to ensure accuracy and stability. Additionally, we further validated the geometric correctness using multiple structural alignments, and incorporated experimental data to enhance confidence of the predicted results.

Phylogenetic trees were constructed using the maximum-likelihood method with aligned protein datasets in MEGA X. Model selection and evaluation were conducted with MrmodelTest 2.0 and ProtTest 2.4, and the JTT + G model was determined to be the most suitable for subsequent analyses. The resulting phylogenetic trees were refined using the iTOL online tool (https://itol.embl.de/, accessed on 22 July 2024). To assess stability of the tree topology, a nonparametric guided analysis was carried out with 1000 bootstrap replicates. For further details on the complete protein sequences, please refer to the Supplementary File.

### 2.2. Experimental Animals and Rearing Conditions

*P. vachelli* individuals (body length 20 ± 0.5 cm) were collected from a standardized aquaculture facility in Neijiang City, Sichuan Province, China. Sixty fish were cultured in two 200-L tanks. All experimental fishes were acclimated for two weeks in a recirculating aquaculture system at Neijiang Normal University (in the same city) prior to experimentation. The rearing conditions were set as follows: water temperature of 28 ± 1 °C, commercial feed (crude protein ≥ 35%) provided twice daily. No abnormal behavior or disease symptoms were observed during the acclimation period.

### 2.3. Pathogen Preparation and Infection Experiment

Our present study utilized a standard strain of *Aeromonas hydrophila* (Ah17), which was obtained from Shanghai Luwei Technology Co. Ltd. This bacterial strain was cultured in semi-solid medium at 37 °C for 8–12 h until reaching a stationary phase (OD600 ≈ 1.0), then resuspended and serially diluted using PBS buffer. The final concentration was verified as 1 × 10^7^ CFU/mL by plate counting. The challenge dose was determined with reference to established studies on hybrid catfish (*Ictalurus punctatus* × *Ictalurus furcatus*) [17], which confirmed that it effectively elicits an immune response without causing excessive mortality. Following injection, all experimental fish were maintained in 100-L recirculating aquaria with water temperature kept at (28 ± 1) °C, dissolved oxygen > 6 mg/L, and ammonia and nitrite levels controlled below 0.05 mg/L and 0.01 mg/L, respectively. During the challenge period, the following indicators were systematically monitored: daily records of mortality, clinical signs such as hemorrhaging, and behavioral abnormalities. *A. hydrophila* was inoculated into LB liquid medium, washed with PBS, and the bacterial solution was adjusted to a concentration of 1 × 10^7^ CFU/mL. Experimental fishes were randomly assigned to an infection group (*n* = 30) and a control group (*n* = 30). The former received an intraperitoneal injection of 20 μL of bacterial solution (1 × 10^7^ CFU/fish), while the control group received an equal volume of sterile PBS. At designated time points post-infection (0, 6, or 12 h), collection of kidney, liver, and gill samples was conducted from three individuals per group per time point, and bacterial loads in tissues were determined via plate counting to evaluate the progression of infection. All tissues were immediately frozen in liquid nitrogen and stored at −80 °C for subsequent RNA extraction and gene expression analysis.

### 2.4. RNA Extraction, cDNA Synthesis, and Quantitative Real-Time PCR (qRT-PCR)

Total RNA was isolated using a TIANGEN RNAprep Pure Tissue Kit according to the manufacturer’s protocol (Tiangen Biotech, Beijing, China). RNA quality was assessed via NanoDrop spectrophotometry and agarose gel electrophoresis. The first-strand cDNAs were synthesized using a FastKing RT Kit (Tiangen Biotech) in a 20-μL reaction as follows: 42 °C for 3 min to remove gDNA; 50 °C for 15 min for reverse transcription; 95 °C for 3 min for inactivation. qRT-PCRs were conducted on a QuantStudio 5 real-time PCR system (Thermo Fisher Scientific, Carlsbad, CA, USA) using SuperReal PreMix Plus under MIQE-compliant cycling conditions (95 °C for 15 min; 40 cycles of [95 °C for 10 s, 60 °C for 32 s]) with a melt-curve analysis to verify specificity. Relative transcript abundance was calculated using the 2^−ΔΔCt^ method, normalizing to *β-actin* as the internal reference [18]. Primer pair sequences are listed in Table 2. All assays were performed in triplicate and repeated independently three times to ensure reproducibility.

### 2.5. Statistical Analysis

Statistical analysis was performed using SPSS 27.0. Data are presented as mean ± standard error of the mean (SEM). For normally distributed variables, independent-samples t-tests were applied for comparisons between two groups [19], while one-way ANOVA was conducted for comparisons among three or more groups [20]. We also performed statistical correction for multiple comparisons across genes, tissues, and time points using the Benjamini–Hochberg procedure to control the false discovery rate (FDR) [21]. Statistical significance was set at *p* < 0.05. Graphs were generated in GraphPad Prism 7.0 [22].

## 3. Results

### 3.1. Genome-Wide Presence of TLR Genes in Various Teleost Species

**Figure 1:**
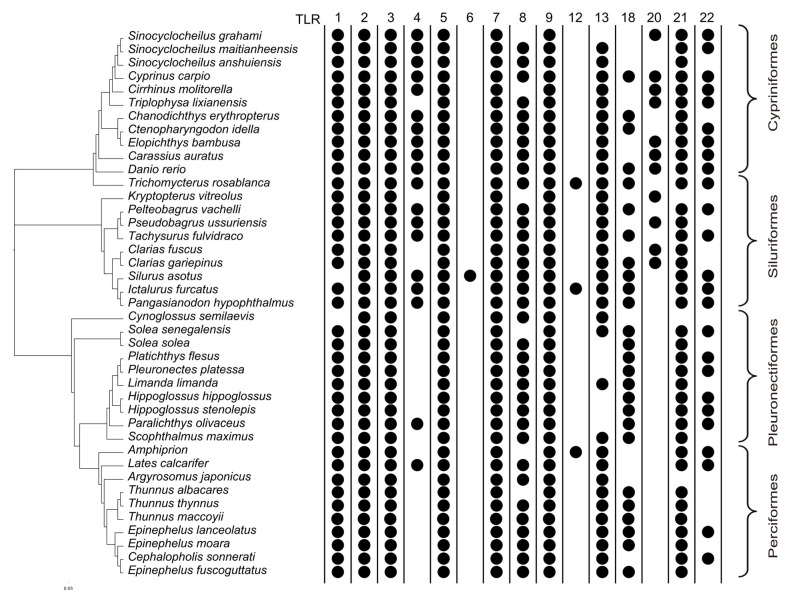
*TLR* members in representative teleost orders. *TLR* genes in 41 fish genomes were analyzed. Each solid circle represents the presence of a *TLR* gene.

Our results illustrate the genome-wide distribution pattern of 15 *TLR* genes (*TLR*1–9, 12, 13, 18, 20–22) across 41 teleost fish species from four major orders (i.e., Cypriniformes, Siluriformes, Perciformes, and Pleuronectiformes). A solid dot graph indicates the presence of these *TLR* gene in each species (Figure 1).

*TLR2*, *TLR3* and *TLR7* are highly conserved across nearly all the examined species, highlighting their critical and ancient roles in the fishes’ innate immunity. Several other *TLR*s, including *TLR1*, *TLR5*, *TLR8* and *TLR13*, are also widely distributed but exhibit certain lineage-specific absence. In contrast, some genes such as *TLR4*, *TLR9*, *TLR18*, *TLR20*, *TLR21* and *TLR22* exhibit a wider distribution, with notable gene loss particularly in species within the Perciformes and Pleuronectiformes orders. Cypriniformes, particularly *Sinocyclocheilus* species, retain a broad *TLR* repertoire, while flatfishes (Pleuronectiformes) generally possess fewer *TLR* genes. Overall, our data reveal both a conserved core set of *TLR* genes and significant variation among diverse lineages, which reflects a dynamic evolutionary history and adaptive diversification of TLRs in various teleost fishes.

### 3.2. Molecular Characterization of TLR Genes in P. vachelli (Pv)

A total of twelve *TLR* genes were identified and characterized in *P. vachelli* (Table 3). The full-length *PvTLR* cDNA sequences ranged from 2089 bp (*TLR4*) to 4456 bp (*TLR9*), with open reading frames (ORFs) ranging from 2370 bp (*TLR2*) to 3264 bp (*TLR8*). The predicted protein products varied in length from 789 amino acids (TLR2) to 1,087 amino acids (TLR8), corresponding to molecular weights from 90.8 kDa to 125.3 kDa. Theoretical isoelectric points (pI) of these TLR proteins ranged from 5.63 (TLR5) to 7.59 (TLR22).

Most TLRs were predicted to possess both signal peptides and transmembrane domains, suggesting good membrane association. However, TLR1 and TLR13 lack signal peptides, and TLR7 lacks a transmembrane domain. Interestingly, two TLRs, including TLR13 and TLR22, contained multiple transmembrane regions, indicating their potential functional diversity. These findings provide valuable molecular data for understanding the structural and functional diversity of TLRs in this species.

### 3.3. Gene Structures of TLRs in P. vachelli

**Figure 2:**
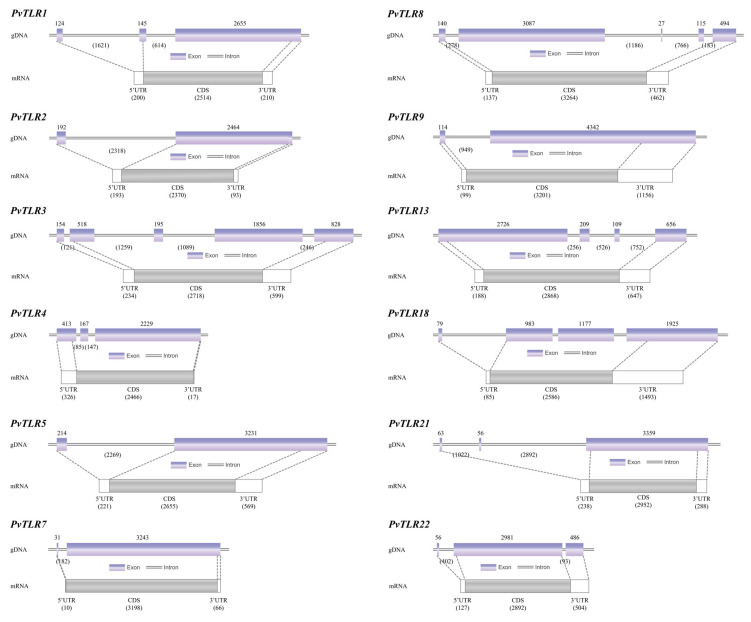
Gene structures of twelve *TLR* genes in *Pelteobagrus vachellii* (*Pv*).

Gene structures of the twelve *PvTLR* genes were analyzed by comparing their genomic DNA (gDNA) and mRNA sequences (Figure 2). All these *TLR* genes exhibited typical exon–intron structures, but with variable numbers and lengths of introns. The coding sequences (CDSs) ranged from 2370 bp (*PvTLR2*) to 3,264 bp (*PvTLR8*), while the 5′ untranslated regions (5′UTRs) and 3′UTRs showed notable variability, suggesting differential regulatory elements. The exon–intron architecture varied significantly, with *PvTLR3*, *PvTLR8* and *PvTLR9* having relatively longer UTRs, indicating complex transcriptional regulation. This structural diversity provides new insights into the evolutionary dynamics and regulatory complexity of *TLR* genes in *P. vachelli*.

### 3.4. Conserved Domain Structures in the PvTLR Proteins

**Figure 3:**
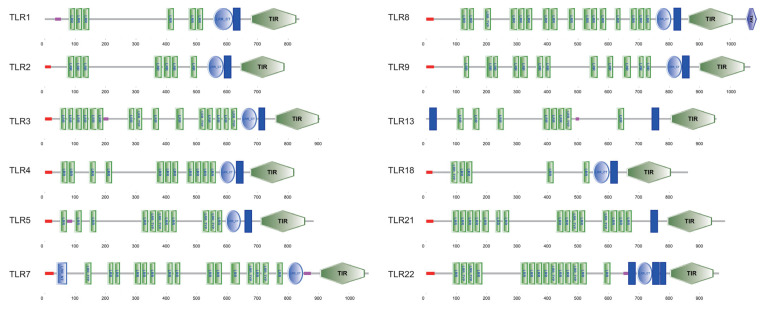
Conserved domain architectures of the twelve PvTLR proteins. Various *TLR* genes contain numerous extracellular leucine-rich repeat C-terminal (LRR-CT), leucine-rich repeat N-terminal (LRR-NT), leucine-rich repeat (LRR) domains, signal peptides, Toll/interleukin-1 receptor (TIR) domain, and a transmembrane domain (TM). The red dots indicate the position of the signal peptide.

Conserved domain architecture of the twelve TLR proteins in *P. vachelli* was analyzed, revealing a typical TLR organization across all the family members (Figure 3). Each TLR protein contains multiple LRR motifs in the extracellular region, responsible for pathogen recognition, as well as a conserved Toll/interleukin-1 receptor (TIR) domain at the C-terminal, which mediates intracellular signaling. Variations in the number and arrangement of LRR motifs were observed among different TLRs, indicating functional diversification. Some TLRs, such as TLR3, TLR8, and TLR9, exhibited an extensive array of LRRs, while others displayed more compact domain structures. These conserved domain patterns highlight the evolutionary conservation of structural features critical for TLR signaling, while also suggesting potential specialization in ligand recognition and immune response pathways.

### 3.5. Predicted Tertiary Structures of the PvTLRs

**Figure 4:**
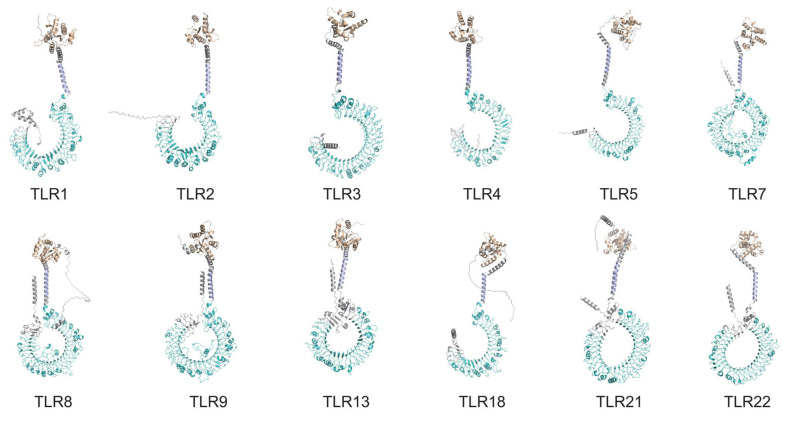
Predicted tertiary structures of the twelve PvTLR proteins.

Predicted 3D structures of the twelve TLRs in *P. vachelli* revealed the common characteristic horseshoe-shaped architecture formed by the LRR domains (Figure 4). These structures consistently exhibit extracellular LRR motifs, a transmembrane region, and an intracellular TIR domain, reflecting their conserved roles in pathogen recognition and signal transduction. Despite the overall structural similarity, subtle variances among the twelve TLRs suggest functional diversification in ligand specificity and immune response modulation.

### 3.6. Collinearity of TLR Genes for Potential Conserved Chromosomal Organization in Teleost Fishes

**Figure 5:**
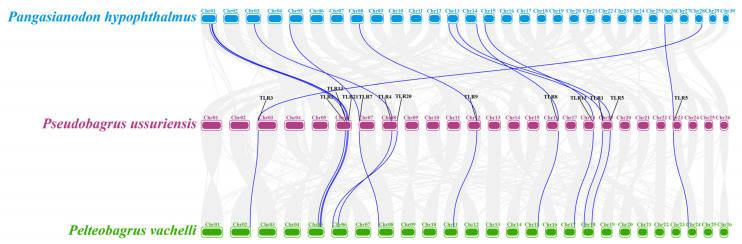
A synteny analysis of *TLR* genes between *P. vachellii* and two related teleost species.

Collinearity analysis between *P. vachelli* and *Pseudobagrus ussuriensi* or *Pangasianodon hypophthalmus* revealed that these *TLR* genes are distributed across conserved chromosomal regions (Figure 5). Although the shared syntenic blocks suggest a high degree of evolutionary conservation of *TLR* genes among the examined species, further functional studies are required to more definitively establish their roles in the innate immune system of various teleost fishes.

### 3.7. Phylogeny Reveals Evolutionary Stability of TLR Subfamilies in Teleost Fishes

**Figure 6:**
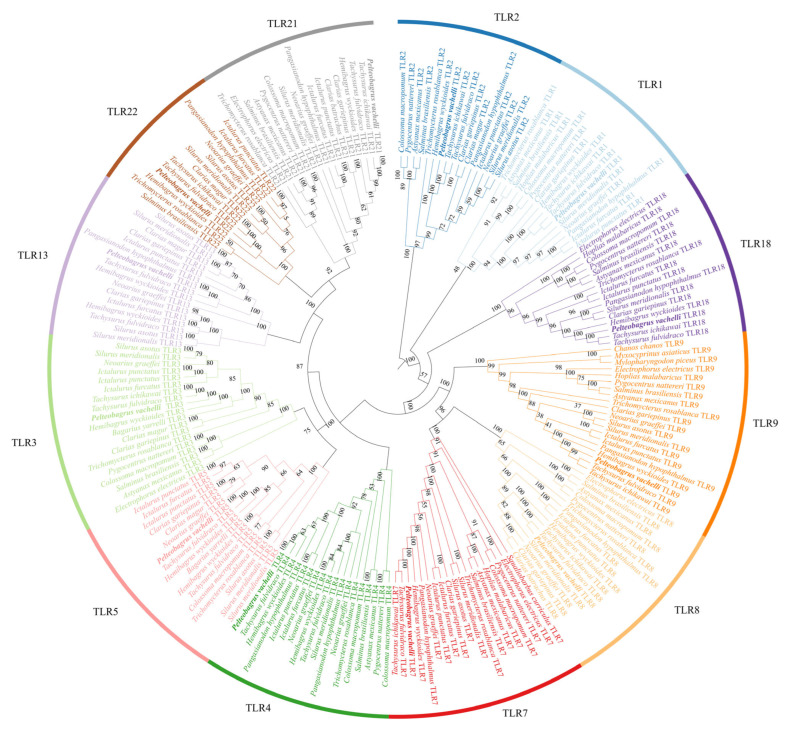
Phylogenetic relationships of *TLR* genes in *P. vachellii* and other representative teleost species.

Phylogenetic analysis of *TLR* genes in *P. vachelli*, alongside other selected teleost species, revealed that each TLR type (including TLR1, TLR2, TLR3, TLR4, TLR5, TLR7, TLR8, TLR9, TLR13, TLR18, TLR21, and TLR22) forms a distinct monophyletic clade with strong (bootstrap support value of 70% or higher) bootstrap support (Figure 6). This clustering pattern highlights the evolutionary conservation of individual TLR subfamily across fish species. Furthermore, the close phylogenetic relationships observed between *P. vachelli* and other Siluriformes species indicate a high degree of sequence similarity and functional conservation within this order. While there is evidence of evolutionary conservation in certain aspects of *TLRs*, the observed lineage-specific gene losses suggest that TLRs have also undergone considerable diversification.

### 3.8. Transcription of TLR Genes in P. vachelli After Infection by A. hydrophila

**Figure 7:**
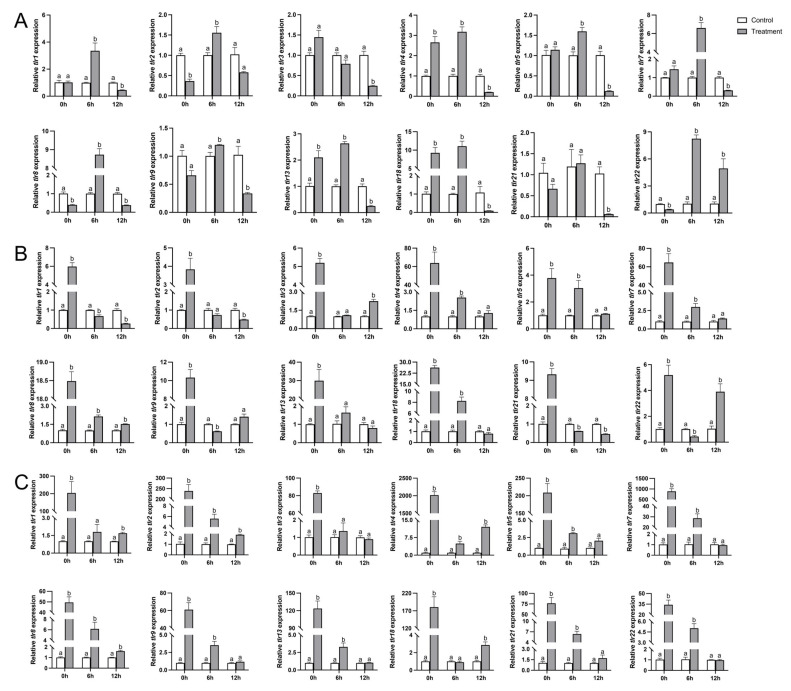
Comparative transcription profiles of the 12 *PvTLR* genes in the kidney (**A**), liver (**B**), and gill (**C**) samples of *P. vachellii*. Groups that differ significantly were indicated by different letters above bars.

As a crucial immunological tissue, the kidney of *P. vachelli* was examined for *TLR* expression dynamics following the bacterial exposure. Our results revealed that infection by exogenous *A. hydrophila* induced significant increases in transcription levels of the 12 *TLR* genes within 12 h (Figure 7), confirming their involvement in pathogen recognition and immune defense.

Upon infection with *A. hydrophila*, the *TLR* gene family exhibited tissue-specific expression profiles in the kidney (Figure 7A), liver (Figure 7B), and gill (Figure 7C). In the kidney, a significant upregulation of most *TLR* genes (nearly all *TLR*s, including *TLR1*, *TLR2*, *TLR4*, *TLR5*, *TLR8*, *TLR9*, *TLR13*, *TLR18* and *TLR22*) were observed (Figure 7A), particularly at 6 and 12 h post-infection, suggesting a rapid and sustained immune response in *A. hydrophila*. The liver also showed notable induction of some *TLR*s (with most *TLR*s showing significant up-regulation, such as *TLR1*, *TLR4*, *TLR5*, *TLR8*, *TLR9*, *TLR18* and *TLR21*), although the peaks of induction were generally advanced (Figure 7B) in comparison with the kidney. *TLR3*, *TLR7* and *TLR13* exhibited moderate to strong activation depending on the time point, indicating a coordinated but tissue-specific response.

The gill tissue displayed the most pronounced and rapid transcriptional response. A dramatic upregulation of *TLR2*, *TLR4*, *TLR7* and *TLR8* was observed, with some genes exceeding a 1000-fold elevation relative to the control group (Figure 7C). Furthermore, *TLR9*, *TLR13* and *TLR21* were also up-regulated, underscoring a critical role of the gill as a mucosal immune barrier. These findings collectively highlight the dynamic and compartmentalized regulation of *TLR* genes in *P. vachelli* after bacterial infection, reflecting their central role in initiating innate immune responses.

## 4. Discussion

Our cross-order survey of 15 *TLR* genes among 41 teleost genomes reveals a two-tier organization of the innate immune sensor repertoire (Figure 1), including a conserved core (*TLR2*, *TLR3* and *TLR7*) retained in nearly all the examined species, and a flexible periphery (e.g., *TLR1*, *TLR5*, *TLR8* and *TLR13* with sporadic absence; *TLR4*, *TLR9*, *TLR18*, *TLR20*, *TLR21* and *TLR22* with patchy order-biased presence). This pattern suggests that the recognition of ubiquitous microbial motifs is safeguarded by deeply conserved receptors, while responses to niche-specific pressures are accommodated by lineage-specific retention, duplication, or loss. Teleost thus appear to balance a stable, essential *TLR* core with a tunable set of auxiliary receptors that track ecological contingency, providing a clear framework for hypothesis-driven investigations of pathogen susceptibility and immune innovation across lineages. For instance, auxiliary receptors like *TLR22* in *Gadus morhua* show species-specific expansion and regulation, suggesting their role as flexible components responding to ecological variations [23].

This study is the first systematic report of 12 *TLR* genes in *P. vachelli*, and their molecular characterization and structural analysis reveal the diverse features of this TLR family. Deduced protein sequences of all identified *TLR* genes exhibit typical TLR-domain characteristics, i.e., extracellular LRR motifs and intracellular TIR domains (Figure 3), which is consistent with the canonical architecture reported for teleost TLRs [8,9]. Notably, different TLR members show marked variation in protein length, isoelectric point, signal peptides, and transmembrane regions (Table 2, Figure 2). For instance, TLR1 and TLR13 lack signal peptides, while TLR7 lacks a transmembrane segment. Similar “non-classical” configurations, such as soluble TLR5 isoforms in salmonids and catfishes, have been shown to alter subcellular localization and signaling properties [15,24]. Gene-structure comparisons reveal diverse exon–intron organizations and significant differences in UTR lengths (Figure 2), suggesting potentially complex post-transcriptional regulation, a mechanism also observed in other fish innate-immune genes [25]. These observations provide a foundation for understanding the structural and functional diversity of the *P. vachelli* TLR repertoire.

Phylogenetic reconstruction places each *P. vachelli* TLR within well-supported monophyletic clades alongside orthologues from other teleost (Figure 6), underscoring their evolutionary conservation. The closest affinities are with TLRs from other Siluriformes species, highlighting sequence conservation within the order [1,26]. Collinearity analysis corroborates a conserved chromosomal distribution pattern (Figure 5), reinforcing the notion that TLRs occupy fundamental and evolutionarily stable positions in the teleost innate-immune systems [1]. However, substantial differences in LRR copy number and arrangement remain (Figure 3); for example, TLR3, TLR8 and TLR9 possess the most extensive LRR regions, which may enhance their capacity to recognize diverse PAMPs [9,27]. Predicted tertiary structures confirm the characteristic horseshoe fold for all TLR proteins (Figure 4), but subtle conformational differences could fine-tune ligand specificity, as previously observed for vertebrate TLRs [8]. Collectively, these structural findings provide crucial insights into the functional diversification and pathogen-recognition strategies of *P. vachelli* TLRs, particularly how their specific expression patterns in different tissues may reflect their functional adaptation to pathogen recognition. Specifically, the distinct structural domains of the TLRs are likely linked to their ability to recognize specific pathogens for a key role in immune responses.

Following *A. hydrophila* infection of *P. vachelli*, the *TLR* gene family exhibited tissue-specific expression profiles in the kidney, liver, and gill (Figure 7). In the kidney, significant upregulation of most *TLR* genes was observed, particularly at 6 and 12 h post-infection (Figure 7A), indicating a rapid and sustained immune response. The liver also showed notable induction of *TLR*s (Figure 7B), although with a generally delayed peak compared to the kidney. *TLR3*, *TLR7*, and *TLR13* displayed moderate to strong activation depending on the time point, suggesting a coordinated but tissue-specific response. In contrast, the gill exhibited the most pronounced and rapid transcriptional response (Figure 7C), with a dramatic up-regulation of *TLR2*, *TLR4*, TLR7, and *TLR8*, some of which even exceeded a 1,000-fold elevation over the control group. *TLR9*, *TLR13*, and *TLR21* were also up-regulated, emphasizing the gill’s critical role as a mucosal immune barrier. As the key mucosal and systemic immune organs in various teleost species, the skin and kidney function as essential frontline barriers and pathogen-processing centers, respectively [28,29]. The rapid *TLR* activation observed in these tissues is consistent with previous findings in common carp (*Cyprinus carpio*), where *TLRs* are similarly up-regulated following exposure to *A. hydrophila* [30]. Notably, the prompt response in both barrier (skin) and lymphoid (kidney) tissues emphasize the coordinated activation of innate immune surveillance systems across different anatomical compartments.

The tissue-specific expression patterns of *TLR*s in *P. vachelli* suggest an adaptation to the different pathogen pressures encountered in aquatic environments. The gill, being the primary organ exposed to environmental pathogens, shows the most pronounced and rapid *TLR* activation. This rapid immune response in the gill highlights its role as a critical mucosal immune barrier, providing the first line of defense against aquatic pathogens. In contrast, the kidney, involved in filtering blood and removing pathogens, exhibits a sustained but slightly delayed *TLR* response, indicating a more regulated immune activation. These tissue-specific patterns reflect how different anatomical compartments are specialized to respond to exogenous pathogens, enhancing the fish’s ability to adapt to the diverse and dynamic pathogen pressures in aquatic ecosystems. This adaptive strategy is crucial for the survival of fish in pathogen-rich environments, where rapid and coordinated immune responses are necessary for effective pathogen detection and clearance.

Furthermore, studies in rainbow trout (*Oncorhynchus mykiss*) and common carp have shown that tissue-specific *TLR* expression patterns were associated with pathogen tropism and entry routes [9]. For example, fish-specific *TLR22* and *TLR23* showed strong induction in the skin during bacterial challenge, supporting the hypothesis that mucosal TLRs may have evolved to specialize in surface defense against aquatic pathogens [10]. Our present results further substantiate this view, as several *P. vachelli TLR*s exhibited marked up-regulation in the gill (Figure 7C), indicating a specialized and rapid mucosal immune response. Taken together, these findings emphasize the importance of TLR-mediated recognition pathways in mounting an effective immune response in diverse fishes and highlight the functional conservation and diversification of the *TLR* gene family across teleost.

## 5. Conclusions

In this study, twelve *TLR* genes were systematically identified and structurally characterized in *Pelteobagrus vachellii*, revealing conserved domain architectures and evolutionary stability across teleost. Their rapid and tissue-specific up-regulation following *Aeromonas hydrophila* infection underscores the essential roles of these TLRs in early immune recognition and the functional diversification of mucosal and systemic immune responses in the darkbarbel catfish. These findings provide valuable genetic resources for the conservation of *TLR* gene diversity in *P. vachellii*, which may inform future breeding strategies for disease resistance. From the aquaculture perspective, the identified *TLR* markers could assist in long-term development of genetically improved strains with enhanced pathogen resilience, ultimately contributing to sustainable production of this commercially important species.

## Figures and Tables

**Table 1:** Species and genomic data acquisition links used in this study.

Species Name	Data Acquisition Link	Species Name	Data Acquisition Link
*Sinocyclocheilus grahami*	https://ftp.ncbi.nlm.nih.gov/genomes/all/GCF/001/515/645/GCF_001515645.1_SAMN03320097.WGS_v1.1/, ((accessed on 20 July 2024))	*Cynoglossus semilaevis*	https://www.ncbi.nlm.nih.gov/datasets/genome/GCF_000523025.1/, (accessed on 20 July 2024)
*Sinocyclocheilus maitianheensis*	https://www.ncbi.nlm.nih.gov/datasets/genome/GCA_045785285.1/, ((accessed on 20 July 2024))	*Solea senegalensis*	https://ftp.ncbi.nlm.nih.gov/genomes/all/GCF/019/176/455/GCF_019176455.1_IFAPA_SoseM_1/, (accessed on 20 July 2024)
*Sinocyclocheilus anshuiensis*	https://ftp.ncbi.nlm.nih.gov/genomes/all/GCF/001/515/605/GCF_001515605.1_SAMN03320099.WGS_v1.1/, ((accessed on 20 July 2024))	*Solea solea*	https://ftp.ncbi.nlm.nih.gov/genomes/all/GCF/958/295/425/GCF_958295425.1_fSolSol10.1/, (accessed on 20 July 2024)
*Cyprinus carpio*	https://ftp.ncbi.nlm.nih.gov/genomes/all/GCF/018/340/385/GCF_018340385.1_ASM1834038v1/, ((accessed on 20 July 2024))	*Platichthys flesus*	https://ftp.ncbi.nlm.nih.gov/genomes/all/GCF/949/316/205/GCF_949316205.1_fPlaFle2.1/, (accessed on 20 July 2024)
*Cirrhinus molitorella*	https://doi.org/10.6084/m9.figshare.24355237, ((accessed on 20 July 2024))	*Pleuronectes platessa*	https://ftp.ncbi.nlm.nih.gov/genomes/all/GCF/947/347/685/GCF_947347685.1_fPlePla1.1/, (accessed on 20 July 2024)
*Triplophysa lixianensis*	https://figshare.com/articles/dataset/Triplophysa_lixianensis_gene_annotation/26326063?file=47765434, ((accessed on 20 July 2024))	*Limanda limanda*	https://ftp.ncbi.nlm.nih.gov/genomes/all/GCF/963/576/545/GCF_963576545.1_fLimLim1.1/, (accessed on 20 July 2024)
*Chanodichthys erythropterus*	https://www.ncbi.nlm.nih.gov/datasets/genome/GCA_024489055.1/, ((accessed on 20 July 2024))	*Hippoglossus hippoglossus*	https://ftp.ncbi.nlm.nih.gov/genomes/all/GCF/009/819/705/GCF_009819705.1_fHipHip1.pri/, (accessed on 20 July 2024)
*Ctenopharyngodon idella*	https://www.ncbi.nlm.nih.gov/datasets/genome/GCA_019924925.1/, ((accessed on 20 July 2024))	*Hippoglossus stenolepis*	https://ftp.ncbi.nlm.nih.gov/genomes/all/GCF/022/539/355/GCF_022539355.2_HSTE1.2/, (accessed on 20 July 2024)
*Elopichthys bambusa*	https://figshare.com/search?q=Elopichthys+bambusa, ((accessed on 20 July 2024))	*Paralichthys olivaceus*	https://ftp.ncbi.nlm.nih.gov/genomes/all/GCF/024/713/975/GCF_024713975.1_ASM2471397v2/, (accessed on 20 July 2024)
*Carassius auratus*	https://ftp.ncbi.nlm.nih.gov/genomes/all/GCF/003/368/295/GCF_003368295.1_ASM336829v1/, (accessed on 20 July 2024)	*Scophthalmus maximus*	https://ftp.ncbi.nlm.nih.gov/genomes/all/GCF/022/379/125/GCF_022379125.1_ASM2237912v1/, (accessed on 20 July 2024)
*Danio rerio*	https://www.ncbi.nlm.nih.gov/datasets/genome/GCF_049306965.1/, (accessed on 20 July 2024)	*Amphiprion*	https://www.ncbi.nlm.nih.gov/datasets/genome/GCF_022539595.1/, (accessed on 20 July 2024)
*Trichomycterus rosablanca*	https://ftp.ncbi.nlm.nih.gov/genomes/all/GCF/030/014/385/GCF_030014385.1_fTriRos1.hap1/, (accessed on 20 July 2024)	*Lates calcarifer*	https://www.ncbi.nlm.nih.gov/datasets/genome/GCF_001640805.2/, (accessed on 20 July 2024)
*Kryptopterus vitreolus*	https://figshare.com/articles/dataset/_b_A_telomere-to-telomere_chromosome-level_genome_of_glass_catfish_b_b_i_Kryptopterus_vitreolus_i_b_/28333385?file=52098524, (accessed on 20 July 2024)	*Argyrosomus japonicus*	https://figshare.com/articles/dataset/Argyrosomus_japonicus_Genome_assembly_and_annotation/20486925, (accessed on 20 July 2024)
*Pelteobagrus vachelli*	https://ftp.ncbi.nlm.nih.gov/genomes/all/GCF/022/655/615/GCF_022655615.1_HZAU_PFXX_2.0/, (accessed on 20 July 2024)	*Thunnus albacares*	https://ftp.ncbi.nlm.nih.gov/genomes/all/GCF/914/725/855/GCF_914725855.1_fThuAlb1.1/, (accessed on 20 July 2024)
*Pseudobagrus ussuriensis*	https://www.ncbi.nlm.nih.gov/datasets/genome/GCA_040256215.1/, (accessed on 20 July 2024)	*Thunnus thynnus*	https://ftp.ncbi.nlm.nih.gov/genomes/all/GCF/963/924/715/GCF_963924715.1_fThuThy2.1/, (accessed on 20 July 2024)
*Tachysurus fulvidraco*	https://ftp.ncbi.nlm.nih.gov/genomes/all/GCF/022/655/615/GCF_022655615.1_HZAU_PFXX_2.0/, (accessed on 20 July 2024)	*Thunnus maccoyii*	https://ftp.ncbi.nlm.nih.gov/genomes/all/GCF/910/596/095/GCF_910596095.1_fThuMac1.1/, (accessed on 20 July 2024)
*Clarias fuscus*	https://figshare.com/articles/dataset/Genome_assembly_and_annotation_information_of_female_i_Clarias_fuscus_i_/26968489, (accessed on 20 July 2024)	*Epinephelus lanceolatus*	https://ftp.ncbi.nlm.nih.gov/genomes/all/GCF/005/281/545/GCF_005281545.1_ASM528154v1/, (accessed on 20 July 2024)
*Clarias gariepinus*	https://ftp.ncbi.nlm.nih.gov/genomes/all/GCF/024/256/425/GCF_024256425.1_CGAR_prim_01v2/, (accessed on 20 July 2024)	*Epinephelus moara*	https://ftp.ncbi.nlm.nih.gov/genomes/all/GCF/006/386/435/GCF_006386435.1_YSFRI_EMoa_1.0/, (accessed on 20 July 2024)
*Silurus asotus*	https://ftp.ncbi.nlm.nih.gov/genomes/all/GCA/024/362/625/GCA_024362625.1_ASM2436262v1/, (accessed on 20 July 2024)	*Cephalopholis sonnerati*	https://figshare.com/articles/dataset/Genome_sequencing_and_assembly_of_the_tomato_hind_Cephalopholis_sonnerati_/27300720, (accessed on 20 July 2024)
*Ictalurus furcatus*	https://ftp.ncbi.nlm.nih.gov/genomes/all/GCF/023/375/685/GCF_023375685.1_Billie_1.0/, (accessed on 20 July 2024)	*Epinephelus fuscoguttatus*	https://ftp.ncbi.nlm.nih.gov/genomes/all/GCF/011/397/635/GCF_011397635.1_E.fuscoguttatus.final_Chr_v1/, (accessed on 20 July 2024)
*Pangasianodon hypophthalmus*	https://ftp.ncbi.nlm.nih.gov/genomes/all/GCF/027/358/585/GCF_027358585.1_fPanHyp1.pri/, (accessed on 20 July 2024)		

**Table 2:** List of the primer pairs used for the qRT-PCR quantitation.

Primer Name	Primer Sequence (5′-3′)	Amplicon (bp)
*β* *-actin F*	GGACCAATCAGACGAAGCGA	105
*β* *-actin R*	TCAGAGTGGCAGCTTAACCG	
*TLR1 F*	TTTGCTAGCCACGAGCTGATG	120
*TLR1 R*	TCTGGCCAGCATTGCCTTTA	
*TLR2 F*	AGCTCCAGTTCGGTAACACG	149
*TLR2 R*	AACTGCCCTGATGGGTTGAG	
*TLR3 F*	ACCTTCTCCGTTTCGACCAC	87
*TLR3 R*	TCGAGCAAGCCGTTTCTGAT	
*TLR4 F*	CAAGGCAGTACTGGAGCCAT	89
*TLR4 R*	AGTTCCAGTATGATGGGCGA	
*TLR5 F*	GAGGCTGACGCTGTTCATCT	136
*TLR5 R*	TGGGCTTCCATCCACGAATC	
*TLR7 F*	GGACGACACTTCCCCAATGT	103
*TLR7 R*	ATTTTTGCAGCTTCGTGCGT	
*TLR8 F*	AGACGTAAGAGCTGGTTGGC	95
*TLR8 R*	GGTCCGCCAGATAAGAGACG	
*TLR9 F*	GCAGATGCTCTGGGTCATGT	129
*TLR9 R*	ATGTTTCCATCGCTGTCCGT	
*TLR13 F*	ATTGTGGTTTGTCTGGCGGT	107
*TLR13 R*	CCCTGGCAGAGGATAGCAAA	
*TLR18 F*	CAGAGCGGGTAACAATCCGT	132
*TLR18 R*	AGCAGGTCTTGAGGGTGGTA	
*TLR21 F*	ACGCTAATGCAGACAGAGTCC	130
*TLR21 R*	GCCATATTTGTCAAAGTGGATGGA	
*TLR22 F*	GACACCAGGGTCTTCTGGCA	142
*TLR22 R*	TCCTCAGCACTCTGCAGATAATTT	

**Table 3:** Sequence details of the 12 *PvTLR* genes.

Gene Name	NCBI Accession Number	Full Length (bp)	ORF (bp)	5′-UTR (bp)	3′-UTR (bp)	Protein Accession Number	Deduced Protein (aa)	Molecular Weight (kDa)	Theoretical pI	Signal Peptide	Transmembrane
*TLR1*	XM_060892079.1	2924	2514	200	210	XP_060748062.1	837	95.3	7.18	No	Yes (1)
*TLR2*	XM_060860014.1	2656	2370	193	93	XP_060715997.1	789	90.8	6.33	Yes	Yes (1)
*TLR3*	XM_060864636.1	3551	2718	234	599	XP_060720619.1	905	103.6	7.07	Yes	Yes (1)
*TLR4*	XM_060872308.1	2466	2089	326	17	XP_060728291.1	821	93.9	6.97	Yes	Yes (1)
*TLR5*	XM_060892327.1	3445	2655	221	569	XP_060748310.1	884	102.0	5.63	Yes	Yes (1)
*TLR7*	XM_060875633.1	3274	3198	10	66	XP_060731616.1	1065	123.0	7.14	Yes	No
*TLR8*	XM_060877162.1	3863	3264	137	462	XP_060733145.1	1087	125.3	7.57	Yes	Yes (1)
*TLR9*	XM_060881940.1	4456	3201	99	1156	XP_060737923.1	1066	122.7	7.15	Yes	Yes (1)
*TLR13*	XM_060870764.1	3703	2868	188	647	XP_060726747.1	955	110.0	6.15	No	Yes (2)
*TLR18*	XM_060870249.1	4164	2586	85	1493	XP_060726232.1	861	98.9	5.84	Yes	Yes (1)
*TLR21*	XM_060870646.1	3476	2952	238	288	XP_060726629.1	983	104.6	7.29	Yes	Yes (1)
*TLR22*	XM_060890409.1	3523	2892	127	504	XP_060746392.1	963	110.7	7.59	Yes	Yes (3)

## Data Availability

The original contributions presented in the study are included in the article. Further inquiries can be directed to the corresponding authors.

## Supplementary Materials

The following supporting information can be downloaded at: https://www.mdpi.com/article/10.3390/biology14121724/s1, Supplementary File: The TLR protein sequences we identi-fied from the 41 species.
